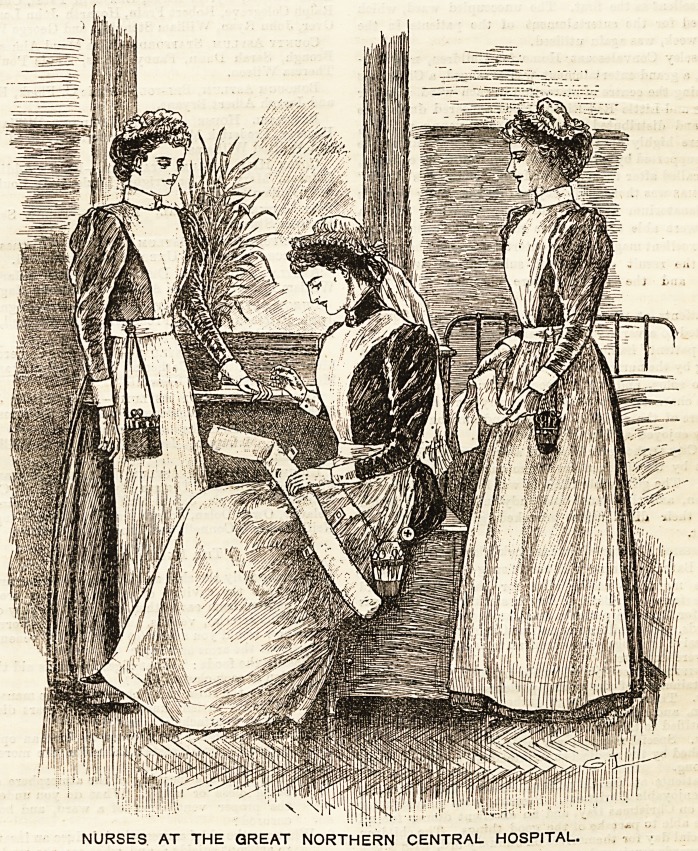# The Hospital Nursing Supplement

**Published:** 1896-01-11

**Authors:** 


"The Hospital" Nursing Mirror.

Being the Extra Nursing Supplement of "The Hospital" Newspaper.

[Contributions for this Supplement should be addressed to the Editor, The Hospital, 428, Strand, London, W.C., and should have the word "Nursing" plainly written in left-hand top corner of the envelope.]

### News from the Nursing World.

### A CHRISTMAS GIFT.

Princess Louise, who is president of the Victoria Hospital for Children at Chelsea, in which she has for so many years taken an active interest, has recently added to many previous gifts that of some interesting coloured pictures for the walls.

### NURSES CONTROLLED BY THE LAITY.

A matter of very grave importance to district nurses was last week brought to our notice in the *British Medical Journal's* correspondence columns. A doctor wrote to inquire as to the advisability of his medical brethren continuing their subscriptions to a district nurse about whom they have not been consulted, as she is "managed by a lay committee, consisting of parsons, &c." Similar cases, we fear, already exist in many other localities, although it is almost impossible to believe that a properly-trained nurse would take any post in which she would not work immediately under the local practitioners. Her services should certainly be requisitioned by them through the medium of the secretary or lady superintendent, who would exercise general control over the establishment. The woman who is content to attend cases to which she is sent by a combined clerical and lay committee without the doctors' knowledge is generally some partially trained person who, useful enough in her way, carries out the work of a paid parish visitor rather than that of a skilled nurse for the sick poor. It would improve the position of true district nurses if lay committees and clergymen would give some other title to the worthy women whose duties they allot independently of either medical supervision or control.

### WHAT WAS THE AGGRAVATION?

A pauper was brought before the magistrate last week for assaulting two officials at Holborn Union four or five days previously. In the interval the unfortunate man had been too ill to leave the infirmary, being phthisical and having, it was admitted, "lost a great deal of blood." It was said that he was drunk when he struck, and afterwards kicked, the porter at the workhouse, and the magistrate sentenced him to four months' imprisonment, without hard labour. In reporting the occurrence, the press does not state whether the patient's disorderly conduct ceased when he was transferred from the hands of the officials who had "got him down and held him," to those of the infirmary nurses, but it is reasonable to believe that it did.

### NURSES IN MINIATURE.

The fancy dress ball given to the children of London by the Lady Mayoress on Tuesday, the 7th inst., was remarkable from the circumstance that of the hundreds of children in fancy costume only one appeared as a nurse. She was a pretty child, correctly dressed, and divided the honours of the most successful design with a young gentleman who appeared as a North American Indian, and whose tattoo marks were wonderfully well done. A group of children wearing the uniforms of the nurses of several of the principal hospitals would prove most effective at a fancy dress ball, and we shall hope to see the idea one day carried out.

### "SUGGESTION AND SYMPATHY."

The proposed constitution of the National Council of Women of Great Britain and Ireland has now been issued in the form of a circular by Mrs. Bedford Fenwick. This states that "donations and subscriptions will be accepted"; that fees shall be proportional to the membership of the society affiliated, the minimum subscription to be £1 per annum. The Council proposes, amongst other objects, to unite all organised societies of women in the United Kingdom, but does not arrogate to itself any power over such organisations "beyond that of suggestion and sympathy." The executive committee is to consist of Lady Henry Somerset, president; Lady Frances Balfour, vice-president; Mrs. Bedford Fenwick, hon. secretary; Mrs. Eva McLaren, foreign corresponding secretary; a treasurer, and others.

### THE SOCIETY OF TRAINED MASSEUSES.

This society is just entering upon its second year of existence, having been established last January "for the purpose of improving the training of and organising an independent examination for competent masseuses." The first of the rules to be observed by members who gain the society's certificate states that no massage is to be undertaken except under medical direction; and the other rules for the guidance of masseuses are equally sound. The next written examination will be held on Friday, January 24th, and the practical on the following day. Arrangements for special instruction in the "Nauheim" treatment have been made, and particulars can be learnt on application to Mrs. Arthur, hon. secretary of S.T.M., 12, Buckingham Street, London, W.C.

### BOLINGBROKE HOUSE HOSPITAL.

A proposal that this home hospital for paying patients at Wandsworth should be taken over by an association, and continue its useful career under new auspices, apparently received a good deal of attention from the guests who assembled at the Christmas party last week. In addition to the private patients, for whom the establishment was originally founded by the influence of Canon Erskine Clarke, the constant friend of the undertaking, accident cases are now admitted free to a special ward. This, in common with the rest of the establishment, was tastefully decorated for the entertainment, and the patients, who seemed exceedingly well cared for, thoroughly enjoyed the programme of music provided for them by the medical staff and the matron.

### A SECOND NURSE NEEDED.

The District Nurse Scheme, put into operation last September at Whitehaven, has already succeeded beyond the most sanguine expectations of its promoters. The increasing needs of the work make the services of a second nurse desirable, and Lord Lonsdale (the mayor of the town) has kindly promised to be responsible for the expenses attendant on this addition to the staff. The selection rests with a committee on which there are three doctors, and it is intended to engage a fully-trained district nurse, at a salary of £30 per annum with board, washing, and uniform. Lady Lonsdale is president of the Whitehaven District Nurse Scheme, and Miss Muncaster hon. secretary, and the simple rules framed by the committee are as commendable as everything else connected with this well-developed institution.

### "ONLY ONE OR TWO."

The nursing of recent fever cases at Longford Infirmary, Ireland, by a trained nurse, has raised a considerable amount of controversy at that institution. Some members of the board expressed dismay at the expenses incurred, apparently considering one guinea a week an exorbitant fee to pay for the nursing of two fever patients. Some unique opinions were expressed at the recent meeting, it being, for instance, remarked (as reported in the local Press) that if there had been only one patient the doctor would have brought in a nurse! The natural inference that the measures adopted by the medical officer had secured isolation, and probably prevented an epidemic, did not appear to occur to the gentlemen, who thought that day and night duty in the fever ward should have been undertaken by some official who would have combined them with her employment in other departments of the workhouse.

### GLASGOW ROYAL INFIRMARY.

The annual New Year's gathering at the Glasgow Royal Infirmary passed off most successfully. It was held in the dispensary hall adjacent to the main building. The chair was taken by the Lord Provost, Sir James Bell, who called attention to the age of the institution, which was, he said, entering on its second century of existence. He congratulated the nurses on the loyal and cordial co-operation they exhibited as well as on their work, and an inspection of their home was made at the conclusion of the meeting.

### ACCIDENTALLY POISONED.

The sad death of Nurse Shaw, of the Glasgow Royal Infirmary, was reported in the *Globe* of the 2nd inst. From the press report it appears that she succumbed to the effects of carbolic acid taken accidentally instead of castor oil. In the face of such a terrible disaster moralising seems misplaced, yet the occurrence cannot fail to set all who hear of it to wonder at medicines and poisons being kept in the same cupboard instead of strictly apart, and the poisons in distinctive bottles. Poor Nurse Shaw is described as a general favourite, and her death has cast a gloom over the institution in which she was valued and beloved. We have strenuously urged the use of distinctive bottles, and have devoted some columns to descriptions of various kinds. Will readers kindly tell us if they have often seen a bottle with a screw stopper used for poisons, and in what institution?

### A NURSE'S RECOGNITION IN FRANCE.

Among the decorations bestowed at the Elysée on New Year's Day was one given to a Sister of Mercy known as Sister Saint-Remi. She received the Cross of the Legion of Honour for having served 39 years in the Military Hospital at Rheims.

### DR. WYNTER BLYTH AT TOYNBEE HALL.

Exceptional advantages appear to fall to the lot of the London Hospital nurses, who have for a long time enjoyed systematic instruction in invalid cookery, in addition to courses of lectures on nursing, elementary, physiology, and anatomy. Those who have passed the examinations annually held in these subjects will now have the privilege of attending a set of lectures on hygiene, which Dr. Wynter Blyth is to give at Toynbee Hall. He has kindly arranged these, at Miss Lückes' suggestion, for an hour, which will enable members of the nursing staff to attend them conveniently.

### ENGLISH NURSES FOR JOHANNESBURG.

Current events give considerable interest to the recent departure of a complete staff of trained nurses for the Johannesburg Hospital, and we feel sure they will be followed by many good wishes to the scene of their future work. Amongst those who have accompanied Miss Young, the new Lady Superintendent, are Nurses Halkett, Butler, MacWilliams, Whiteman, Eleanor Smith, Kingsbury, Covey, Archer, Chester, Rees, Whear, Griffin, Waller, Wardell, Binney, Couche, Hollis, Temple, Cox, Daly, E. Smith, Tobias, Janson, King, Timpe, Eger, and Carter. They started on Saturday, 4th inst., in the ss. Trojan, *viâ* Southampton, and formed a party of thirty fully-trained, certificated, and experienced nurses. The hospital which has secured such services may well be congratulated, and we trust that our sisters, who have gone out with a determination to do credit to their several training schools, will enjoy health and happiness in their new country. They will doubtless be heartily welcomed by the medical staff by whose advice they have been secured for the colony. It may be well to mention that connected with the various hospitals in South Africa there is a considerable staff of nurses, the Kimberley Hospital having twenty-five and ten probationers, the matron being Miss Nacher, late matron at the Poplar Hospital. The new Somerset Hospital at Cape Town has twenty-three or more nurses; Grahamstown, eight; Petermaritzburg, six; Port Elizabeth, eight, besides several others.

### SHORT ITEMS.

The Loughborough Board of Guardians recently passed a courteous vote of thanks to the Ladies' Committee, whose voluntary work had benefited so many persons in the Union.—The monthly "At Homes," held for the staff of the Nurses' Co-operation, 8, New Cavendish Street, continue to be very well attended and greatly appreciated.—The Visiting Committee have recommended the Halifax Guardians to engage an experienced nurse for children under three years of age in the new nursery.—At a meeting, with Lady Radnor in the chair, it was resolved that an association should be formed to provide a district nurse for the villages between Bodenham and Stratford Toney.

### Lectures on Nursing.

By a Superintendent of Nurses

### IV.—CARE OF BEDDING, BEDS, BED-PANS, AND LAVATORIES.

A nurse should take care to keep her ward as tidy as possible, as the general appearance depends so much on attention to details in this respect.

The beds should look as uniform as possible, the quilt being put on so that its centre is in the middle of the bed, the same applying to the sheets, which should be turned down a uniform length. Very little attention seems to be paid to this matter. In some hospitals I have seen several empty beds together, and in one the pillow has been quite covered and the sheet turned down over the pillow only, the next bed having the sheet turned down so as to show half the pillow, &c. This gives the ward anything but a trim look.

Linen soiled in the slightest should never be dried in the ward, thereby not only causing a disagreeable smell, but rendering the air impure. Great care should be taken to protect the mattress. It is, to to say the least of it, a very disgusting thing for it to get saturated with urine. Every new patient should be supplied with a mackintosh under the sheet or draw-sheet. These mackintoshes, when not in use, should be rolled, and not folded, as the latter causes them to wear out very quickly in the folds, and then they are useless. It is a good plan to roll them up with old linen to prevent their sticking. Sapolio is the best thing to use in scrubbing them, and they should be well dried before being put away.

In your zeal for tidiness, do not worry your patients, or cause them to feel as if pinned down. On the other hand, remember there are few things more uncomfortable to a patient than an untidy bed, with the clothes all over the place, the pillows hot and damp, and the linen generally moist. Very often just straightening the under sheet first, and tucking it in tightly, then shaking the pillow (not over the patient), turning the cool side up, and making the top sheet and blanket straight, will afford unbounded relief, and cause the patient to doze off into refreshing slumber.

Before putting on clean sheets while the patient is in bed they should be aired and kept warm; and in placing the under one on, it should, as a rule, be rolled lengthwise side by side with the soiled one, which, freed from the bolster, should be rolled up. An assistant nurse should very gently turn the patient on his side ever so little, while the sheets are unrolled under him. He can then be put back on the clean sheet, which is drawn straight by the assistant nurse, and the soiled one removed. If the patient cannot be moved from side to side, one nurse will have to push the soiled sheet out on the sound side, whilst on the affected side the nurse passes her hand under the patient and helps to draw the sheet through. A top sheet should be put on before the soiled one is removed. In piling up pillows to support a patient, do not arrange them so as to cause the head to be thrown forward. See that the small of the back is well supported, and in raising him to enable him to drink comfortably, place your hand behind his pillow and under his shoulders, so as not to hurt his neck.

If a patient has been lying long in his bed, it is a very good plan to give him a change by putting him into another one. To do this, draw a bed alongside the one he has been occupying, and move him very gently on to it, either by sliding or by lifting him by the sheet, or by a blanket placed under him, which he should retain until he is nice and warm.

Never allow a patient to get up for any purpose, unless you know he is permitted to do so, and if he has permission to use the night chair, be careful and see he is well wrapped up, and has his feet on something warm.

Before emptying a bed-pan used by a patient, look carefully and quickly at the motion. If you see anything at all unusual in it, show it to the sister, as evacuations always afford important indications as to the condition of the patient. You may find them looking like clay, indicating insufficiency of bile; or black, indicating blood; or it may be the result of the patient taking iron. They may be very hard, indicating costiveness; or loose, showing a tendency to diarrhœa. Medicines of various kinds cause the motions to appear of different colours. You may sometimes see mucus or pus in the stools, so always notice evacuations; and report the fact, also, if they have an offensive smell.

And here let me say a word as to the giving of the bed-pan. I know full well how trying this duty is at first—to girls who have been brought up in refined homes—but remember you are attending sick people, and rendering help to such. Oh! how many nurses try to shirk this piece of work. I once knew a "ministering angel," who looked so sweet, yet, rather than ask her for the bed-pan, patients would suffer ever so much, and wait till another nurse came on duty. Think of the misery of being forbidden to get up, and yet having to ask an unwilling attendant to render such a service. I have known nurses pretend not to hear such requests. Put yourself in the patient's place. "There is much in the daily life of a nurse," says one who speaks with authority, "to blunt her sensibilities. Familiarity with suffering takes from its reality, the perpetual presence of death robs it of half its mystery, and awe; seek then as an antidote to cultivate a spirit of reverence in the presence of these realities, encouraging in yourself and in your patients a veneration for that masterpiece of mechanism, the human body, so you will preserve a spirit of modesty and decency in them and in yourself, and whatever you see or have to do by being met in that spirit will pass you by unsullied.' You may not only pass through with your womanhood unscathed, but you may incalculably influence and elevate those under your care.

Never let vessels containing urine stand about near the beds of your patients, for not only is it a most unpleasant sight, but its vicinity to a patient must have a sickening effect upon him. Urine should always be thrown away directly it has been passed, unless required for testing or other purposes.

Then as to the lavatories. You cannot be too particular about these. Not only should the w.c. pans be kept scrupulously free from impurities, but every day some chloride of lime should be thrown down the sluices, and the plugs pulled, and, indeed, it is well to pull the plug frequently, day and night, that the water in trap and pan may not become impregnated with the foul gas arising in the soil pipe.

Care should be taken that all typhoid excreta, properly disinfected, should be thrown down one sluice, and that bed-pans used should have a distinguishing mark upon them, so that they are not used for other patients.

### Death in Our Ranks.

We much regret to announce the death of Nurse Catherine Boor, 4, Adelphi Terrace, Salford, Manchester, on New Year's Day, after a few days' illness, from typhoid fever. She was a Queen's nurse, and much esteemed by all who knew her.

### Nurses and their Competitors.

ALL who have watched the progress made during recent years in the art of nursing, and in the position occupied by those who have taken up that work, see well enough that the nursing calling has become a little world in itself—its education, its mode of life, its aims, its hopes, all being peculiar to itself, and distinguishing it from that greater world within which and for the sake of which it has its being.

By hard work, by long training, and by much self-sacrifice, "trained nurses" have reached a position far higher than some thirty years ago it would have been thought possible for women to attain who were engaged in work which was always looked upon as disagreeable, and was by many thought to verge upon the menial; yet all the time this nursing world, this self-made and self-constituted profession, has been threatened with a danger. It has been hedged round by no legal protection; it has not been able even to define its terms of membership; its members have rejoiced in the title of "trained nurse," yet what constituted a training has never been decided; and all around it there has been a fringe consisting of those who certainly were nurses, in that they lived by nursing, but who certainly were not "trained" as "training" has come to be understood. Gradually, as the position of the real nurses has improved, the number of their imitators has increased, and not only have they increased in number, but they have improved greatly in efficiency, so that the difference between the two classes in the public eye has ceased to be so great as it used to be. Years ago the distinction between the new trained nurse and the Mrs. Gamp of old was obvious to everyone, and it was easy enough for the nursing world to say, and to impress upon the public, who was without and who within the pale. But now it is different. Not only do nursing manuals abound, so that it is easy for any intelligent hanger-on at a hospital to gain such superficial knowledge as to enable her in manner, in dress, and in method, to pose before the public as the modern nurse, but there are hundreds of women who actually have been trained —in some sort of way or in some sort of hospital or another—and who thus can honestly speak of themselves as "trained nurses," and yet are outside the pale in consequence of their hospital or their degree of training not being recognized by the leading schools. What, then, is to be done with these women? It is easy to answer leave them alone, maintain the standard of recognized nursing at the highest possible pitch, and trust to obtaining some time some legal recognition of the profession which will keep out interlopers. This is the line of action which has been to a large extent adopted by the leaders, and it is clear enough that it has resulted in a wonderful elevation of the whole tone of the nursing world. But, on the other hand, it is also clear that while it has produced a splendid *corps d'élite* for the service of the wealthy and of hospitals and workhouses, it has tended to fly over the heads of great masses of people who cannot afford the more expensively trained nurses. Here comes another side of the question. It is useless for nurses to shut their eyes to the demands of the public in the matter, for, whether they bury their heads in the sand or not, the public not only ask for a cheaper article but actually obtain it from the fringe, the hitherto unrecognised fringe, of the nursing world. Again, it is easy to say that this is all very wrong, and that the employment of these untrained, or at least unrecognised, nurses is to be in every way deprecated. But the public answers, "You have killed our dear old Mrs. Gamp, you will not come for less than a couple of pounds a week, and what are we to do?" It is impossible to deny that in this there is some reason, and more especially when we bear in mind a point which has been far too little considered in discussions on the matter, viz., that, after all, the half-trained nurse, the nurse trained at a cottage hospital, or the woman who has worked under a sister at a fever hospital as a second assistant during an epidemic, poorly as she may compare with the fully-trained nurse, is yet a great improvement on the woman she displaces—the old type of kindly, fussy ignorance and dirt.

Let us look at facts. We will not enter too minutely into the standard of training required by too many of the nursing homes or institutes, for it is notorious enough that in many of them nurses are employed who would not be recognised by others. Then, as regards the R.B.N.A., we would not like to vouch for all the hospitals where its members profess to have been trained being recognised as training schools. Again, there are the hundreds of "probationers" who are being turned into nurses at infirmaries, fever hospitals, cottage hospitals, and other unrecognised places, who, after their period of probation is over, are somehow earning a living as nurses. Lastly, we must consider the fact that the country is gradually being overspread under influential patronage, and under the direction of very active propagandists, by a system of nursing which is confessedly different and inferior to what is recognised as "trained."

Again then we ask, what is to be done with these women? Will the nursing profession recognise the urgency of the public demand for something less than a complete training, and hold out the hand of friendship to a second grade of nurses?

We have recently drawn attention to the establishment of a system of village nursing in the county of Nottingham, a system according to which women of intelligence and good character who are willing to live with and among the people of a small country village, to accept wages much the same as the labourers themselves, to go from cottage to cottage doing the work of a district nurse, and to attend any confinement which may require their assistance, are at the expense of the association sent to Plaistow, where, under the tuition of Sister Catharine, they receive a six or nine months' training in general nursing, and are taught midwifery in a practical manner, so as to be able to pass the examination of the London Obstetrical Society. It is proposed to call them county nurses. This is a system which has obtained a firm foothold, and the association by which it is promoted is already affiliated to the Queen Victoria Jubilee Institute. Moreover, the report received regarding the work done by these nurses is satisfactory. They are not midwives alone, nor are they trained nurses, but they are able to render great service in nursing the poor, and they occupy a field which is not and cannot be touched by nurses of a more complete and expensive training.

Are, then, trained nurses to stand aloof and to allow this great mass of women, who are *bonâ fide* engaged in nursing work, to remain outside the nursing profession, deprived of the stimulus and the good influence, and even the discipline, which they would obtain from association in their work with those ladies who have devoted their energies so usefully to the development of the nursing art? Or, shall we not rather recognize two grades of nurses—one fully-trained, the highly accomplished nurse, such as is now being turned out from a few training schools; the other, the cheaper but still useful nurse, who after a moderate amount of teaching undertakes the simpler work required in a workaday world? This is the important question which we put forward for consideration, for undoubtedly the danger is imminent that notwithstanding, nay—partly in consequence of—the yearly increasing efficiency of fully-trained nurses, much of the work of nursing will gradually pass into the hands of cheaper but entirely undisciplined women who, from being excluded from the profession, will hold themselves free from its duties, its obligations, and its high traditions.

### Dress and Uniforms.

By a Matron and Superintendent of Nurses.

### GREAT NORTHERN CENTRAL HOSPITAL.

The busy little group before us represents a sister, staff nurse, and probationer of the Great Northern Central Hospital. The pretty, bright wards with their polished floors and spacious accommodation are in pleasing contrast to the smoke and dirt of the streets outside, and strike the eye of extremely good, and has a very clean, fresh appearance, relieving the somewhat sombre hue of the dress. A small mob-shaped cap of white cambric completes the costume. It is trimmed wi.h a row of Coventry frilling, edged with lace, which is gophered and drawn in with a thread on the reverse side, to fit the shape of the head. Two long ends

NURSES AT THE GREAT NORTHERN CENTRAL HOSPITAL.

a visitor forcibly on entering. In peaceful harmony with the surroundings are the nurses, whom we have here the pleasure of depicting. The sister, who occupies a seat in the centre and is apparently engrossed in demonstrating the art of splint padding, is attired in a dress of navy blue drillette. It is a washing material, and in consequence the most suitable for wear in a sick ward. The white linen apron, of ample width, is provided with a bib which is high enough to encircle the neck, at the back of which it terminates in straps, which cross and fasten beneath the band at the waist. The effect is fall at the back and give a very graceful finish to the coiffure The nurse and probationer wear light blue twilled Galatea dresses alike. The bodice and skirt are made perfectly plain, the former buttoning down the front. The skirt is made full from the waist-band, and just off the ground. An apron with a bib of similar shape to that worn by the sister covers the dress, and neat turn-down cuffs and collars make a becoming finish to the wrists and neck. The caps are distinguished from that worn by the sister in not having ends, otherwise the shape is the same. Plain Coventry frilling trimming differentiates the probationer from the staff nurse who wears lace edging like the sister.

### Christmas Entertainments.

The nurses' entertainment at the London Temperance Hospital was a most successful and enjoyable one. Music and recitations were followed by Grossmith's amusing musical sketch, "Cups and Saucers," which was cleverly given by Mr. and Mrs. Alfred Stalman. "My Uncle's Will" was as much enjoyed by those amongst the audience to whom the comedietta is an old favourite, as by others who heard the clever dialogue for the first time. The second part of the programme was as excellent as the first. The unoccupied ward, which had served for the entertainment of the patients in the previous week, was again utilised.

At Moseley Convalescent Home for Children, near Birmingham, a grand entertainment was arranged, a Christmas tree forming the centre of attraction in each ward. Father Christmas and Little Red Riding Hood appeared during the evening and distributed presents to the young patients, which were highly appreciated. One of the wards at the Home is supported by contributions from well-to-do children, and it is called after them "The Moseley Children's Ward."

Christmas was thoroughly enjoyed by the young patients in the Sanatorium at Middlesbrough, of whom about a hundred were able to appreciate the huge Christmas tree and the excellent magic lantern entertainment. The supply of toys, the result of a special subscription, was most generous, and the children were charmed with their treasures.

The patients at the National Hospital, Queen's Square, were treated to a variety and amusing entertainment in the large out-patients' hall. The programme commenced with a carol sung by the nurses, followed by "Nazareth," violin and violoncello solos, and various songs. An extravaganza, in which the characters from the nursery rhymes were depicted by members of the resident medical and nursing staff, was immensely enjoyed and was well performed.

At the City Orthopædic Hospital a very happy Christmas was spent by the children, for whom a magnificent tree was provided. The gifts with which it was laden gave immense satisfaction, and the Punch and Judy entertainment which followed their distribution provoked great delight and merriment.

A succession of entertainments took place at the United Hospital, Bath, beginning with carols sung by the nurses on Christmas morning, useful gifts for the patients, and seasonable fare. On Boxing Day a smoking concert was arranged for the male patients and carols were sung in the other wards. The children had a fine Christmas tree and for two successive evenings some excellent tableaux and "Waxworks" were exhibited.

A magnificent tree lighted by electricity was prepared for the entertainment of the young patients at Dundee Royal Infirmary. There was a large gathering of friends of the institution, and some pleasant speeches were made, all of which testified to the high reputation enjoyed by the Royal Infirmary. Special mention was made of the valuable work accomplished by the medical superintendent, and the matron, Mrs. Strong.

The patients at the City Hospital, Parkhill, Liverpool, spent an enjoyable Christmas. Presents were provided for everyone on Christmas Day, and an excellent dinner for all who were able to partake of the good things. The children had a special day for themselves on January 3rd, with a tea, and afterwards music and games.

Christmas at the National Sanatorium, Bournemouth, was made as bright as possible for the patients by many kind friends. A concert was got up for New Year's Day, and on January 2nd a Christmas tree and entertainment were provided, when useful gifts were distributed to all.

The wards of the new Passmore Edwards Cottage Hospital, Wood Green, were prettily decorated for Christmas, and on January 4th a concert and Christmas tree were much enjoyed by the patients. Miss Warren, the matron, superintended the festivities, at which many of the medical officer and members of the committee were present.

### Medico-Psychological Association.

At the November examinations for the certificate of proficiency in nursing granted by the Medico-Psychological Association the following candidates were successful:—

Warwick County Asylum.—Harriett Buswell, Ada Cowland, Ruth Coates, Jane Jones, Harriet Kirk, Gertrude Ladbrook, and Fanny E. Robbins.

North Riding Asylum, Clifton, York.—Angela Brooks, Alice Cooper, Sylvia Large, Elizabeth Yates, Charles Best, Ralph Colegrave, Robert Frain, Hogarth John Lord, Walter Over, John Ryan, William Stenning, and George White.

County Asylum, Stafford.—Alice Maud Aimes, Mary A. Brough, Sarah Dunn, Fanny Dukes, Annie Poulston, and Theresa Wilson.

Borough Asylum, Bristol.—Annie C. Flook, Eva Smith, and Joseph Albert Bryant.

Bethnall House Asylum, London.—William Robert Hegerty, Frederick Meek, John Morrison, Alfred W. Street, and Wilfred Williams.

Coton Hill Asylum, Stafford.—Bertha Addison, Sarah Crinean, Mary A. Cartwright, Lizzie Hall, Louisa Smith, and William Emery.

St. Luke's Hospital, London.—Fanny Sexton and Florence Stuart Zerfass.

Halliford House Asylum, Sunbury.—Thomas Burrows.

Moorcroft Asylum, Uxbridge.—Alfred Hall.

Argyle and Bute District Asylum.—Mary Peters, William Ratray, William Allan, and Malcolm Haggart.

Burgh Asylum, Paisley.—Charlotte Gray, Agnes Kröger, Jessie Paterson, Thomas Freebairn, John Mitchell, and John D. MacLeod.

Perth District Asylum.—Margaret Cameron, Grace Munro, Jane Stuart, Catherine Ward, John Fraser, James McKenzie, and Robert Stewart.

Roxburgh District Asylum.—Alice Keith, Jane Ann McFarlane, Charles Henderson, and John Thomson.

Stirling District Asylum.—Christina Muirhead, Catherine McIntyre, Isa Colinson, and William John Dalgleish.

Richmond District Asylum, Dublin.—Thomas Brennan, Frederick Brunton, Thomas Byrne, William Culverwell, Patrick Jos. Doyle, James Duffy, Joseph Doran, William Delaney, Owen Dolan, Joseph Hanley, James Langan, Peter Neill, James O'Connell, and Thomas W. Graham.

### The Examination Paper.

1. Describe fully the skin and its parts: What are its uses?
2. What are the cavities of the trunk, how are they separated from each other, and what do they contain?
3. Describe arterial, venous, and capillary hæmorrhage, and how would you arrest (*a*) arterial, (*b*) venous bleeding from the arms and legs?
4. Classify the foods: What digestive fluids aid the absorption of each class of food?
5. What are the rules to be observed in the management of an insane patient known to have heart disease, and subject to attacks of syncope?
6. In what way would you proceed to form an opinion of a person's mental state? Mention the more common forms of insanity?
7. Describe fully the meaning of the atmosphere of a ward being close or stuffy. What do you understand by the proper ventilation of a ward, and how is this ensured?
8. What are the chief causes which induce an insane patient to attempt suicide; and what are the modes of self-destruction usually adopted by (*a*) males, (*b*) females?
9. What is an hallucination? Mention the chief forms, and explain why they are so important as symptoms of insanity.
10. What is a bedsore? Is it a preventible disease? Describe fully how you would guard against its formation?
11. In (*a*) epileptic, (*b*) paralytic, and (*c*) senile cases, what dangers might occur, and what precautions should be adopted?
12. Mention the special points to be observed in the bathing of patients?

### The Winter Exhibitions.

Exhibition of Spanish Art, New Gallery, Regent Street.

The chief interest of the exhibition of Spanish art at the New Gallery centres round the works of two painters, Velasquez and Murillo, and, if for them alone, the show is altogether beyond the average. Throughout the collection of over 900 pictures there are but 50 from the master hands of these two great artists, but, as we have said, these 50 examples stand out in a trenchant manner. Not that Spain has not produced other artists, for indeed there are works hanging on these walls which would do credit to any country—Claudio Coello's for instance, and Vincenzo Carducho's. The "Portrait of the Painter" by the latter is a specimen of exceptionally good work. This formerly was in the Louis Philippe collection. Carducho was Court painter to Philip III. and Philip IV. of Spain, and had a literary, as well as an artistic, fame; his pictures hang in all the galleries of Castile and other large Spanish cities. Zurbaron, perhaps the best known Spanish painter after Velasquez and Murillo, has some beautiful specimens here of his art. For instance, in the West Gallery, in an all too inconspicuous place, hangs his half-length figure of a "Lady in the Character of St. Elizabeth of Hungary." A curious charm this picture has upon us, not only for the actual prettiness of the little lady's face, but for the exquisite manner in which it is painted. Some twenty years ago or more this picture was exhibited at the Royal Academy among the works of old masters, and many who saw it then felt the fascinating hold which it possesses.

St. Elizabeth is represented in a blue and gold embroidered dress, with a sceptre in her hand and a crown upon her head; behind her is a deep red curtain, forming a harmonious background, and some poor people are on the right of the portrait. Zurbaron, who was a close imitator of Caravaggio, was called the "Spanish Caravaggio"; he also was Court painter to Philip IV., the great patron of art of those days. Perhaps his studies of a more sombre nature are more representative of the man, and his pictures of "St. Stephen" and "Christ in Glory" show him better as he is generally known than does the sweet and winsome St. Elizabeth. A large size study of "A Magdalen" (No. 82) in the West Gallery is a typical instance of the artist's particular style, which is ever characterised by a serious treatment of serious subjects.

But all Spanish art was of a serious nature in its earlier period, and representative then of a thoughtful and a religious people. The directors of the Gallery are to be congratulated on the excellence of the quality and of the quantity of Murillo's works in this collection. Although Murillo was a pupil of Velasquez, we find small expression in his canvasses of the admiration he bore his great master; he was no imitator, and his genius was certainly not modelled on the lines of any other artists or of Van Dyck, whom he also studied under. Many of Murillo's works here exhibited are well known to us, either through the medium of other exhibitions or through engravings. But their beauty comes ever freshly upon us. A central position on the south side of the West Gallery is given to his "Flower Girl" (No. 35), a picture which belongs to Dulwich College, to which gallery it was bequeathed. This shows a young girl seated on a stone bench, dressed in a yellowish bodice and oversleeve. The mellow colouring thoroughout is exquisite, and it would be hard to overpraise the whole scheme and working of the picture. Near by hangs another example of Murillo's art in a somewhat opposite direction. "A Portrait of Don Andres De Andrade" has rather the effect of a nightmare upon us. Grim, austerely he stands, full length before us, in a dark doublet with slashed sleeves and knee-breeches. Whether through juxtaposition with the smiling flower girl or from its own merits, the grimness of this portrait is forced upon us. Further along on the same wall the artist's well-known Virgin as "Mater Purissima" hangs below his, perhaps even better known, "Virgin and Child" (No. 40). This picture is supposed to be the one referred to by Murillo in his will, which he painted for a weaver in discharge of "nine veras of satin." "The Infant Christ Asleep" is a lovely study of a lovely child, and is full of the peace of which the painter was so eminent an exposer. Another picture from the Dulwich Collection, "Two Peasant Boys," is in the same gallery on the north side; where also hangs "The Marriage of the Blessed Virgin" and "St. Joseph." Turning to the North Gallery we find "Ecce Homo" (No. 139), "Christ after the Flagellation," and a series of five pictures hanging on the central line of the north side, the subject of which is "The Prodigal Son Receiving His Portion." The canvas somehow does not show Murillo at his best, and we ra ther gladly turn to his wonderful studies of "Ecce Homo" which are hanging near, and to "The Veil of Veronica," one of the most perfect specimens from this artist's brush.

Velasquez is shown to us in the New Gallery, as indeed he is most generally shown, as a portrait painter—a painter of people more than a painter of subjects. It is a curious thing to consider that the European fame of Velasquez is of a fairly modern date, for he was not at all adequately appreciated during his lifetime; and still more curious it is that he is entirely unrepresented in the galleries of his native city of Seville. We have some grand specimens of his works at this exhibition, the earliest of which we have any authentic record being the famous "Water Carrier," hanging in the north room; and a wonderful piece of work this is. But more actually pleasing are the artist's studies, which occur at intervals throughout the collection, of "Mariana of Austria, second wife of Philip IV."; and each time these portraits occur they strike one more and more for the exquisite beauty of their vivid colouring. Philip IV.'s wife had a remarkably attractive face, with the complexion of a wild rose. Velasquez made a great study of this pure, delicate skin. Then his portraits of her husband, in the various stages of his life, are monumental pieces of work. No. 43, a three-quarter length figure, is a masterpiece of colouring. Tender and exquisite, too, is No. 44, called "Child and Serving Man," a portrait study from life, painted in all the fresh exuberance of Valasquez's most artistic power. Some other studies of children place this painter in the foremost rank of child portrait painters; it is barely possible to find a more lovely study of childhood than his picture of Philip IV.'s eldest son and his dwarf. "Pope Pius X." is also a remarkable picture, of which there are two studies in the Gallery.

We have lingered intentionally over the pictures of these two master painters for, as has been said already, the works of all others at this exhibition fall into the background by their side. But many among those of lesser glory are well worth studying, and to appreciate the exhibition as it deserves, more than one visit should be paid to it. The modern exhibits, other than those of Goya, are not very attractive, and, indeed, do not show well by the side of the greater painters represented; but it is interesting to see the art of Spain as it has been, and as it is.

A Collection of Portraits of the 17th and 18th Centuries, The Dowdeswell Galleries, 160, New Bond Street.

The delightful little exhibition now being held at the Dowdeswell Galleries is distinguished more for the quality than for the quantity of the portraits. Each painting—and there are but twenty-two in all—is a perfect specimen of the master hand of such artists as Miereveldt, Cornellius

Janssens, Gerard Van Houthorst, Sir Godfrey Kneller, James Northcote, and Sir Thomas Lawrence, &c. Of the Miereveldts there are no less than six examples, each painted in with the faithfulness as to detail and the general breadth of treatment which characterises the work of this Dutch genius. Seen by themselves, or closely contrasted with others, Miereveldt's technique is in its way unique. One portrait in this collection, No. 6, "A Lady," one of a pair with "A Gentleman," is a study of old age in its happiest aspect. Calm and peaceful the old lady sits there before us, in her furs and her lace, the very embodiment of matronly propriety. Cornellius Janssens' portraits of Lord and Lady Waterpark are two charming studies. Janssens lived in Vandyk's time, and one sees a slight impression of his mannerism on the lesser artist. Life-like and powerful is Sir Godfrey Kneller's "Mr. Whitley," No. 9 in the collection. Indeed, English portrait art finds some of its highest expressions in the example just referred to, and in James Northcote's "Floral Offering" and Sir Thomas Lawrence's "Lady Peel." Of these three pictures it is impossible to speak too highly; each, too, examples of very different methods of work. The strength and breadth of Sir Godfrey Kneller's, with its deep warmth of colouring, and the exquisite delicacy and refinement of Sir Thomas Lawrence's, in close proximity, are shown in forcible contrast, each so striking, and each so different. We owe something to Messrs. Dowdeswell for this choice collection, which is unfortunately thrown open to the public for a short time only.

It will interest many of our readers to hear that Mrs. Ernest Hart is exhibiting a collection of her pastel studies, entitled "Glories of the Sky and Sea in the Far East," at the above galleries.

### Appointments.

Llanelly Hospital.—Miss McGowan has been appointed Matron of the Llanelly Hospital. She has held a similar post at Fleetwood Cottage Hospital for two and a half years, and holds good testimonials. Miss McGowan takes many kind wishes for her future success to Llanelly, and we congratulate her on her appointment.

Miss S. A. Warburton has been appointed Matron to the City of London Infirmary, Bow Road, subject to the approval of the Local Government Board. Miss Warburton received her training at the London Hospital, and has since held the post of Matron Superintendent at Her Majesty's Hospital, Stepney Causeway, for over six years. Her testimonials are excellent, and the Guardians are to be congratulated on their choice. We cordially wish Miss Warburton all success in her new work.

### Presentations.

On New Year's Day the nurses of the Fir Vale Infirmary, Sheffield, presented their matron (Miss Thomlinson) with a handsomely chased silver card case.

Mrs. Mathias, of the Royal United Hospital, Bath, was presented on Christmas Day with a beautiful silver teapot from the nurses, silver teaspoons from the servants, and some old china from the resident staff.

The nurses of the County Lunatic Asylum, Thorpe, Norwich, have recently, on her birthday, presented to the matron, Miss Hamer, a gold bracelet, as a small token of their esteem.

### Wants and Workers.

[The attention of correspondents is directed to the fact that "Helps in Sickness and to Health" (Scientific Press, 428, Strand) will enable them promptly to find the most suitable accommodation for difficult or special cases.]

Can any of your readers help to find a school or home for a little boy with infantile paralysis in legs and back? Stated to be an incurable case. Address A. R., 57, Lambeth Road, Kennington, S.E.

### Sleep and Sleeplessness.

By a Night Sister.

"How many thousands of my poorer subjects are at this hour asleep!" Thus moralised a wakeful monarch; had he lived in the present hospital days, the knowledge that many hundreds, if not thousands, of patients and nurses were also at this hour awake might perhaps have afforded him some consolation. Sleep, with its refreshing power and soothing influence, we all seek and desire.

It is rare, but possible, for anyone to lie awake all night in cases of acute pain, of distress from difficult breathing, with brain trouble, &c. There is no mistaking the wan, tired expression of those who greet the morning with glad relief after having, as they feel, lived years in one night's suffering. These are not the patients who complain that they have not slept. They will say they have had a bad night, but the overwhelming truth of the fact has subdued within them any desire to exaggerate. Such patients deserve our entire sympathy, and although much may be done by good nursing to help them through the long hours, still it is for such cases as these that the physician's skill is demanded to help by means of drugs. For people who lie awake a good deal to say that they have not slept all night is not rare; in fact this occurs constantly. Healthy people who fall asleep soon after putting their heads on the pillow and do not wake again till called scarcely appreciate the wearisomeness of an opposite experience. Those who manage to sleep during some part of the night do not, as a rule, get sufficient sympathy; although they seem irritable and tired, they have not the careworn expression produced by a really bad night.

By gradual degrees and by varied efforts the patient may be induced to acquire again a healthy habit of sleep, the refreshment intended for man by the merciful provision of nature, the loss of which is more or less due to man's interference with nature's laws. Arranging the bed in a comfortable manner, the application of a little extra warmth, such as a hot-water bottle or an additional blanket, a short conversation to turn the thoughts into a pleasant direction, will often do much towards procuring for a patient really refreshing sleep. A glass of boiling milk, cooled to the taste from a fresh syphon of soda-water, is an excellent remedy for persons lying awake at night. A cup of hot milk or beef tea, or good cup of cocoa taken after getting into bed, has often proved useful for those who complain of being awake during the early hours of the night, as it is quite likely that their sleeplessness is due either to an anæmic condition or malnutrition, or may be to worrying upon business or other matters. Hypnotics or sleeping draughts are of value, but should never be given except by a medical man, nor should a dose ever be repeated because the patient still lies awake, or the nurse considers that the dose is insufficient; repetition might prove dangerous.

### Royal British Nurses' Association.

The secretary of the Royal British Nurses' Association desires to call the attention of members and others to the series of lectures on "The Nursing of Nervous Diseases," now in course, by Dr. Colman, on Thursday afternoons, at three p.m., at 17, Old Cavendish Street. The first, dealing with the affections of the peripheral nerves and spinal cord, was delivered on the 9th inst., the subject of the lecture on the 16th inst. is "The Cerebral Hemispheres and General Symptoms of Brain Disease." The quarterly meeting of the General Council was held on Friday, 10th inst., at which H.R.H. the President was present.

### Everybody's Opinion.

[Correspondence on all subjects is invited, but we cannot in any way be responsible for the opinions expressed by our correspondents. No communications can be entertained if the name and address of the correspondent is not given, or unless one side of the paper only be written on.]

### NURSES' UNIFORM.

"POLICY No. 3,862" writes: I have read with great interest the various discussions about uniform, but beg to say I think the suggestions which appeared in last week's issue the most sensible of all. I think the suggestion made by "Policy No. 3,162" a very excellent one, as there are a great many very good nurses who are not policy holders and again, there are a great many who began to provide for themselves in other ways before the R.N.P.F. was founded. I quite think with "Policy Holder 47" that a bonnet and cloak designed by our Princess quite out of the question, as most all hospitals and institutions have their own particular uniform.

### MALE NURSES.

JOHN F. RUBBINS writes: Seeing the letter in a recent number of THE HOSPITAL, and being a male nurse, I wish to say a few words on the subject. Myself I see no reason why male nurses should not be tried and prove successful. I was for two years a probationer in a hospital for special diseases, and we were trained under a female staff nurse and a sister, and received exactly the same training as the opposite sex, and I should have been most pleased to have been able to enter a general hospital, so as to have got a thorough training, but at once found there was no institution in England where male nurses were admitted. All engaged in nursing must admit that there are a great number of cases which are most unfit for female nurses. Conversing on the matter to a secretary of a hospital, he informed me that male nurses were a failure; but with proper management and discipline I think they would work successfully.

### "PRACTICAL ASPECTS OF A NURSE'S LIFE." HINTS TO BEGINNERS.

"A COUNTRY MATRON" writes: I have read with much pleasure "Lectures on Nursing," by a Superintendent of Nurses, and thoroughly agree with her advice on keeping up home interests. It is with regret that I see opposite advice given by Sister Grace. Nurses should keep their minds bright and their sympathies warm with interests outside their hospital life if they are to bring these minds and sympathies effectively to work for their patients. They are only women, and must also receive if they are to keep on giving. Nothing is so satisfying as a patient's gratitude, but sick people are naturally self-absorbed; we expect them to be so. The interests of hospital life run in a groove, and small things become abnormally important. It is only by keeping in contact with the larger world that we can take a just view of our surroundings, our fellow-workers, and, perhaps, especially ourselves. The dissatisfied nurses I have seen were those who had lost touch with their outside friends, and had lost their individuality in becoming a nurse. After years of a nursing life they awoke to the knowledge of the barrier their own selfish indifference and neglect had raised between themselves and those who should have been dearest to them. It is not self-sacrifice, but selfishness, which makes a nurse so absorbed in her new life as to neglect her home ties, and it does not ennoble but impoverishes her character to shut herself up in her little hospital world. Inside her ward she must live entirely for her patients, but outside let her leave it all behind as much as possible, including her uniform. In so doing she will be refreshed in every way, and become a more efficient nurse and a truer woman.

### New Registration of Nurses.

WE have received the following from the secretary of the Parliamentary Bills Committee of the British Medical Association:—

At the meeting of the British Medical Association, held in London in August last, a resolution was passed to the effect that, "In the opinion of this meeting it is expedient that an Act of Parliament should, as soon as possible, be passed, providing for the registration and education of medical, surgical, and obstetric nurses; and the Council of this Association are therefore requested to consider this matter, and to take such measures as may seem to them advisable to obtain such legislation." This resolution was subsequently referred by the Council to the Parliamentary Bills Committee of the Association. With a view to ascertaining the wishes and views of the nursing bodies and training schools on the subject, a conference will be held at the offices of the Association, 429, Strand, on Tuesday, January 14th, at three p.m., under the presidency of the chairman of the Parliamentary Bills Committee, Mr. Ernest Hart, at which many of the leading institutions interested in nursing have signified their intention of sending representatives. All nursing and training bodies desiring to take part in the conference are requested to communicate their wish to Mr. Francis Fowke, General Secretary, at 429, Strand.

### Notes and Queries.

The contents of the Editor's Letter-box have now reached such unwieldy proportions that it has become necessary to establish a hard and fast rule regarding Answers to Correspondents. In future, all questions requiring replies will continue to be answered in this column without any fee. If an answer is required by letter, a fee of half-a-crown must be enclosed with the note containing the enquiry. We are always pleased to help our numerous correspondents to the fullest extent, and we can trust them to sympathise in the overwhelming amount of writing which makes the new rules a necessity. Every communication must be accompanied by the writer's name and address, otherwise it will receive no attention.

### Queries.

(69) *Workhouse Nursing.*—Will you kindly state how I can get information on workhouse nursing? What is the difference between ordinary and workhouse infirmaries, and what are the prospects of advancement after a training in the latter?—*M. E. P.*

(70) *District Nurse.*—Please tell me where a thoroughly-trained, certificated nurse who holds a midwifery certificate could get a post as district nurse.—*E. M.*

(71) *Indian Nursing Service.*—Is there any hospital in London where training is particularly suitable as a preparation to nursing in India?—*N. C.*

(72) *Probationer.*—Am I too old to enter a hospital? My age is 18.—*C. M. F.*

(73) *Cottage Hospital.*—Will some one kindly give me information as to the routine and management of a cottage hospital?—*Frances.*

(74) *Army Nursing.*—Can you give me information about applying for a post as army nurse for Africa or Turkey?—*G. C.*

### Answers.

(69) *Workhouse Nursing* (*M. E. P.*).—You can get information by applying to the hon. secretary of the Workhouse Infirmary Nursing Association, 6, Adam Street, London, W.C. We presume by "ordinary" you mean general infirmaries, which are the same as general hospitals. Workhouse infirmaries are under the Poor Law, and there is plenty of good work to be done in them. If you enter one of these be sure and find out first whether systematic instruction, lectures, and certificates are given. Of course, probationers in these infirmaries have the prospect of being made charge nurses eventually if they prove efficient. There are also posts as night superintendents, assistant matrons, &c., which are much sought after. The demand for trained nurses in workhouse infirmaries at present exceeds the supply.

(70) *District Nurse* (*E. M.*).—Look in our advertisement columns this week. Have you had training in district work? You can get information about the Queen's Jubilee Nurses by applying to Miss Peter, St. Katherine's Hospital, Regent's Park, London. They do district nursing amongst the poor.

(71) *Indian Nursing Service* (*N. C.*).—No hospital provides special training for India. A well-trained nurse will find no difficulty in accommodating herself to the different conditions she will encounter.

(72) *Probationer* (*C. M. F.*).—As we have often pointed out to our readers, it is injudicious to begin a nurse's career until the age of 21, and this age is too young except for a small children's or cottage hospital as a preliminary to a further three years' training at a good general hospital.

(73) *Cottage Hospital* (*Frances*).—Write to the Scientific Press, 428, Strand, for a copy of Burdett's "Cottage Hospitals," which will be published immediately, and contains all the information you require.

(74) *Army Nursing* (*G. C.*).—See "The Hospital Nursing Supplement," p. lxxiv., for November 30th, 1895. The only nurses sent on service are "Her Majesty's Nursing Sisters." If your training makes you eligible to become one of these, you can obtain a form of application at the War Office.

### For Reading to the Sick.

**Motto.**

If every year we would root out one vice we should sooner become perfect men.—*Thomas à Kempis.*

**Verses.**

Go with the spiritual life, the higher volition and action,
With the great girdle of God, go and encompass the earth!
Not for the gain of the gold, for the getting, the hoarding, the having,
But for the joy of the deed—but for the beauty to do.
—*Clough.*

Thou mightest have been one of us,
Cleaving the storm and fire;
Aspiring through faith to the glorious,
Higher and ever higher;
Till the world of storms look tremendous
Far down, like a smitten lyre.
—*George Macdonald.*

A man's best things are nearest him,
Lie close about his feet;
It is the distant and the dim
That we are sick to greet;
For flowers that grow our feet beneath
We struggle and aspire;
Our hearts must die, except they breathe
The air of fresh desire.
Thou who canst think as well as feel,
Mount from the earth! Aspire! aspire!
—*Wordsworth.*

**Reading.**

Although in the system of the natural life of man time past can never be recalled, there is such a thing in the economy of grace as "redeeming the time." When our works are done with a full faith in the pardoning, restoring ove of Christ, with an ardent, enthusiastic desire to please Him, and yield Him all the little, miserable tribute that we can, when the consciousness of past falls and neglected opportunities redoubles our energy—when, like Peter, plunging into the water to meet his Lord, we burn with desire to show Him that we love Him more than those who have not wounded Him so deeply—then, in those days of vigorous Christian impulse, we redeem the time, and God restores to us the years which the locust of self-indulgence or irreligious toil has eaten.

Lift up, then, the hands that hang down, and the feeble knees! God gives us more days still—gives them surely that they may be redeemed, not that they may follow their predecessors into the dark ravine of unreclaimed opportunities. If He lightens our darkness once again, a fresh dawn to-morrow will suffuse itself over the face of nature. . . Why should it not be a dawn of spiritual life, and hope, and energy in thy heart—a dawn which shall shine more and more unto that perfect day, the day of consummated holiness and endless enjoyment?—"Thoughts on Personal Religion," *Dean Goulburn.*

### Where to Go.

The Children's Fancy Dress Ball at the Mansion House on the 7th inst., given by the Lady Mayoress, was largely attended. Many of the costumes were excellent in design, but the effect of the ball was marred by want of adequate organisation. Instead of roping off the floor of the Egyptian Hall and giving it up entirely to the children, the adults, of whom there were a great many present, crowded into the space and interfered much with the dancing. The procession, too, was anything but a success, owing to the same cause. Instead of the children being marshalled and marched past the Lord Mayor and Lady Mayoress with ample space, they were huddled together, and it was impossible to see the costumes, thus spoiling a pretty demonstration. This must have interfered with the enjoyment of the children as it did with that of the spectators. The band of the Coldstream Guards discoursed excellent music, and there were marionettes, a Punch and Judy show, and other amusements for the children, all of which were greatly appreciated.

### The Book World for Women and Nurses.

[We invite Correspondence, Criticism, Enquiries, and Notes on Books likely to interest Women and Nurses. Address, Editor, The Hospital (Nurses' Book World), 428, Strand, W.C.]

"The Men of the Moss Hags." By S. R. Crockett. (Isbister and Co. London. 1895.)

The space at our disposal renders it impossible to give more than an outline of the plot of Mr. Crockett's new novel. The story is one of adventure; the scene is laid in Scotland during the closing years of the seventeenth century; and there is abundant evidence in every page that the author has thoroughly mastered the history of those times. It is certain that he does not exaggerate the iniquity of the system under which Scotland was governed during the reign of Charles II., and we do not think the picture which he gives us of the cruelty with which the servants of the Crown carried out the severe orders of their employers, or of the heroism of their victims, is in any way overdrawn. So long as he is treading the heather, and painting the scenery of the martyrland, Mr. Crockett is at his best. The moorlands are all round us. The hills lie quiet and silent in the haze, or the black thunderclouds brood over them and the lightning flashes among the glens. We see the black peat mosses, among which the wanderers lie hidden, and the bosky hillsides, down which the brown streamlets rush, under the shadow of the mountain ash. In all this Mr. Crockett is original and fresh. But there are parts of the volume before us which smell of the lamp, and make us feel as if the author had sat down at his desk with his cloud of witnesses, and his "Hind Let Loose" and a pile of other books which most of us have read. We do not accuse Mr. Crockett of having read up his subject for the purpose of writing about it. His work is too good to have been produced by the common methods of the hack-writer. But while he has been refreshing his own memory on topics with which he has himself been long familar, he has forgotten how many readers have been over the same ground.

Our Secret Friends and Foes. By Percy Faraday Frankland, F.R.S. (London: Society for Promoting Christian Knowledge.)

From the title of the work the casual reader might imagine that he was taking up a novel or a romance, and although this would not exactly be the case, as far as interest is concerned, it is not so far off the truth as might be expected. The subject dealt with is purely scientific, but the story of bacteria as told by Mr. Frankland is quite as enthralling and far more interesting than the majority of novels which cover the railway bookstalls. The book is divided into seven sections, the first of which deals with the general description of bacteria and their habits. The crucial question, whether bacteria are animals or vegetables is, however, studiously avoided. The next section treats of bacteria in air, and the third of those in water. In the fourth section the use of bacteria in the economy of nature is duly emphasised, and the question of the fermentation of alcoholic beverages fully described. The uses of bacteria in furthering the growth of both animals and vegetables is also pointed out. The part that bacteria play in the causation of disease will be of special interest to readers of The Hospital, and the last chapter is of no little importance, inasmuch as it deals with the destructive influence of light on the growth of micro-organisms, and hence in the prevention of disease. If even a few of the intelligent public can follow the simple reasoning of this chapter, the object of the Society in publishing this work will have been more than gained.

## Figures and Tables

**Figure f1:**